# High-Pressure Injection Injuries of the Hand: A Report of Three Cases Presenting With Acute Compartment Syndrome

**DOI:** 10.7759/cureus.72786

**Published:** 2024-10-31

**Authors:** José Miguel Azevedo, Gonçalo Tomé, Dmitry Shelepenko, Inês Catalão, Susana Pinheiro, Sara Ramos

**Affiliations:** 1 Department of Plastic and Reconstructive Surgery and Burns Unit, Coimbra Local Health Unit, Coimbra, PRT

**Keywords:** compartment syndrome, cytotoxicity, hand trauma, high-pressure injection injuries, surgical debridement

## Abstract

High-pressure injection injuries of the hand occur after contact with the nozzle of a high-pressure injecting system such as a paint gun or air compressor, usually on the non-dominant hand of industrial laborers. The severity and real extent of damage in high-pressure injection injuries are often hidden behind a small punctiform wound at initial presentation and are generally underestimated. High-pressure injected material spreads into the tendon sheath, along neurovascular bundles and fascial planes, resulting in neurovascular compromise and acute compartment syndrome. Some products are extremely cytotoxic and can lead to chemical damage and tissue necrosis, with the potential for secondary infection. We present three cases admitted to our department requiring urgent evaluation and treatment. All cases had a distinct mechanism of injury and different materials injected; nevertheless, all had a similar clinical presentation. Symptoms included severe pain, swelling, tenderness, a punctiform wound, and hypoesthesia of the fingers, consistent with acute compartment syndrome, which constitutes a surgical emergency. The first case involves a 49-year-old man who presented to the emergency department after an accidental injection of graphite lubricant oil into the palm of his right hand. The second case is a 57-year-old man who sustained a high-pressure injury from an injection of an industrial car wash product in the thenar region of his right hand. The third case is a 37-year-old man who presented after an accidental high-pressure injection of an industrial anti-corrosive primer into the left palm at the metacarpophalangeal level of the index finger. The three patients underwent immediate surgical exploration for the decompression of the thenar and mid-palmar compartments, copious saline irrigation, and wide debridement of the foreign material and devitalized tissues plus carpal tunnel release. This approach in combination with antibiotics and tetanus prophylaxis has achieved satisfactory results with significant clinical improvement and early discharge without neurovascular compromise. Prompt diagnosis, early surgical intervention, and postoperative intensive physiotherapy are essential for hand salvage and function restoration in this type of injury.

## Introduction

High-pressure injection injuries of the hand occur after contact with the nozzle of a high-pressure injecting system such as a paint gun or air compressor. The pressures that are present within these systems can range from 100 psi for a grease gun to 10000 psi in hydraulic systems. It usually occurs on the non-dominant hand of industrial laborers working with chemicals such as paints, lubricants, or fuels. Accidents commonly occur while cleaning injection devices and generally affect young males [1-3]. The incidence is 1/600 hand traumatisms, accounting for 1-4 cases per year in large centers [4]. The index finger, the thumb, and the palm of the hand are the most commonly affected areas [4-7].

The direct mechanical impact of the high-pressure product generates compression within a confined compartment space. Furthermore, these products often have cytotoxic properties. These combined can have devastating consequences if not diagnosed and treated promptly [3]. The severity and real extent of damage are often hidden behind a small and frequently painless punctiform wound at initial presentation, leading to underestimation and delayed treatment. Pain may be absent early, but the affected digit gradually swells and becomes painful and numb, and vascular compromise commonly follows [8].

We report three cases admitted and treated in our department, each with distinct injury mechanisms and type of material injected. Nevertheless, all had a similar clinical presentation characterized by acute compartment syndrome requiring immediate surgical intervention.

## Case presentation

Case 1

**Figure 1:**
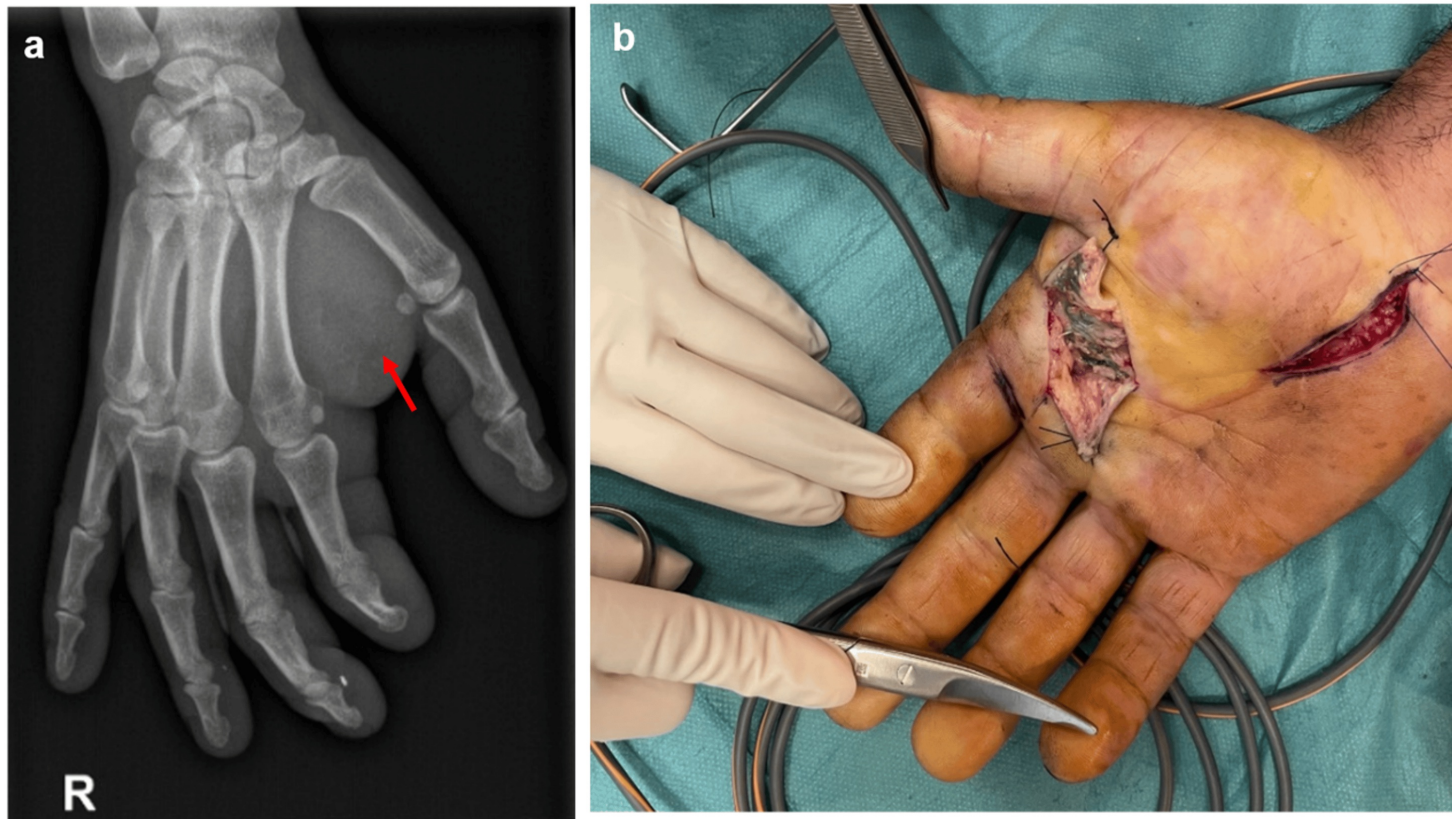
Case 1 (a) Admission X-ray of the right hand showing soft tissue edema (arrow). The material is radiolucent. (b) Intraoperative clinical findings showing extensive graphite infiltration in both subcutaneous and deep planes at the palmar metacarpophalangeal level of the index finger. A carpal tunnel release incision can be seen.

A 49-year-old automobile mechanic presented to the emergency department with severe pain and swelling in his right hand, 24 hours after an accidental injection of graphite lubricant oil into his palm while using a pressure gun at work. On clinical examination, the hand was swollen and tense, with a punctiform wound present at the injection site (palmar metacarpophalangeal level of the index finger). The patient complained of finger numbness in the median nerve territory and limited flexion of the fingers. On the X-ray, only soft tissue edema was present (Figure 1a). Immediate surgical exploration revealed extensive graphite infiltration in both subcutaneous and deep planes, involving intrinsic muscles and flexor tendons (Figure 1b). Surgical debridement, abundant irrigation, product removal, and carpal tunnel release were performed. A suction drain was placed before closing the wound primarily. The patient showed significant clinical improvement and was discharged two days postoperatively with analgesia and prophylactic antibiotics. There was a good primary wound healing. One year later, the patient still experienced mild numbness and paresthesia in the index finger and thenar region, occasional pain triggered by certain movements, and slight grip weakness despite maintaining a normal range of motion. 

Case 2

**Figure 2:**
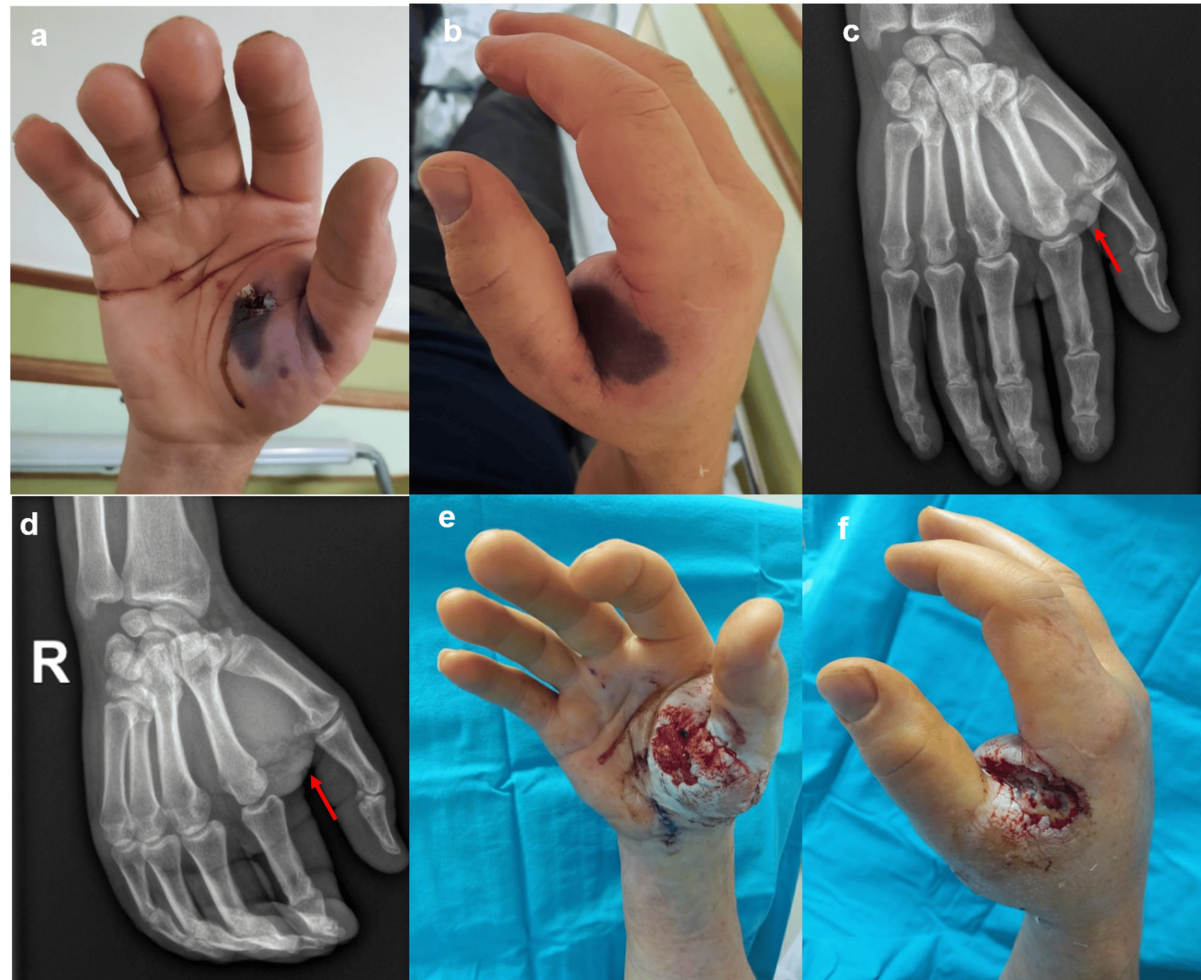
Case 2 (a, b) Admission clinical presentation showing a pinpoint wound with brownish fluid drainage and skin compromise in the thenar region. (c, d) Admission X-ray of the right hand revealing soft tissue edema and subcutaneous emphysema (arrow). The injected material is radiolucent and therefore cannot be seen. (e, f) Later clinical presentation after surgical debridement and wound dressing care, showing lesions in the thenar region that required skin grafting.

A 57-year-old man presented to the emergency department with pain and numbness in the first and second fingers of his right hand after accidentally injecting an industrial car rim cleaning chemical into the thenar region. He was immediately referred to our hospital. In addition to thumb and index finger hypoesthesia, on clinical examination, he had a pinpoint wound with brownish fluid drainage and skin compromise in the thenar region (Figure 2a, 2b). Radiography revealed soft tissue edema and subcutaneous emphysema (Figure 2c, 2d). Immediate surgical exploration showed skin necrosis and soft tissue liquefaction in the thenar region, extending through the first web space to the dorsum of the hand, affecting neurovascular structures and thenar muscles. Decompressive fasciotomy of the thenar compartment, extensive washing, debridement of devitalized tissues, and carpal tunnel release were performed. The patient had a favorable clinical course and was discharged on the third postoperative day. He required three revision surgeries for further debridement (Figure 2e, 2f), skin grafting in the thenar region, and sensory nerve reconstruction of the radial digital nerve of the thumb and first web space common digital nerve with a sural nerve graft. At the one-year follow-up, the patient exhibited substantial improvement in strength and function after physical therapy, along with gradual recovery of finger sensitivity, currently only experiencing numbness in the pulp of the thumb.

Case 3

**Figure 3:**
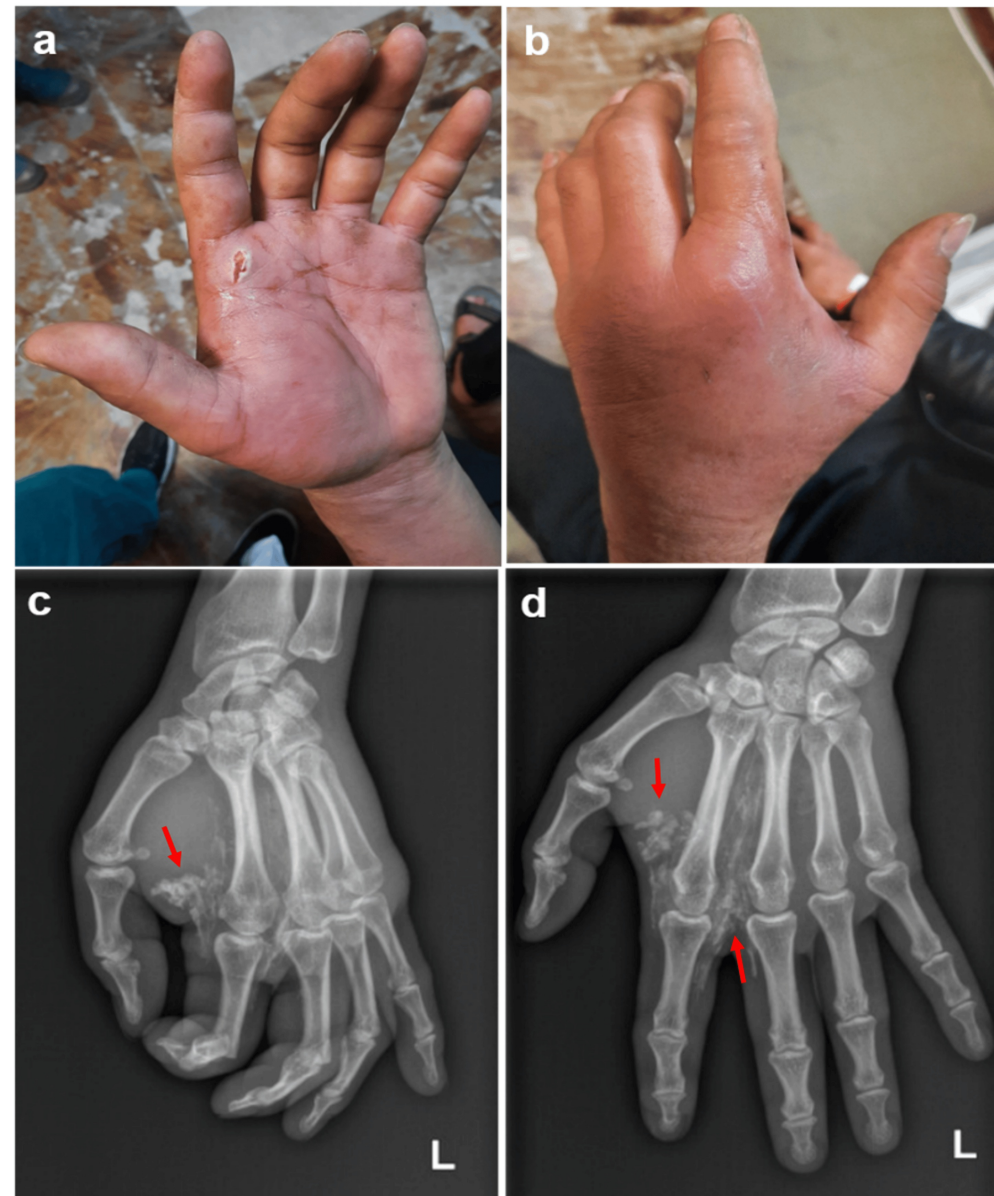
Case 3 (a, b) Clinical presentation with a small punctiform wound at the injection site, located at the palmar metacarpophalangeal level of the index finger. (c, d) Admission X-ray of the left hand showing extensive infiltration of dense material (arrows) in the thenar and mid-palmar regions, extending into the second and third fingers.

A 37-year-old right-handed industrial worker from a wind turbine factory presented to the emergency department reporting excruciating pain, swelling, and numbness in the first three fingers of the left hand, two days after a work accident involving his paint gun. This resulted from the accidental high-pressure injection of an industrial anti-corrosive product (primer) into the palm at the metacarpophalangeal level of the index finger (Figure 3a, 3b). Initially, there were only minimal complaints, but the symptoms worsened significantly over the following hours, with pain and edema becoming unbearable 48 hours later, resulting in functional and sensory impairments. Physical examination revealed signs of hypoperfusion of the index finger characterized by pallor and prolonged capillary refill time. The product was radiopaque, and X-rays showed extensive infiltration of dense material in the thenar and mid-palmar regions, with extension into the second and third fingers (Figure 3c, 3d).

**Figure 4:**
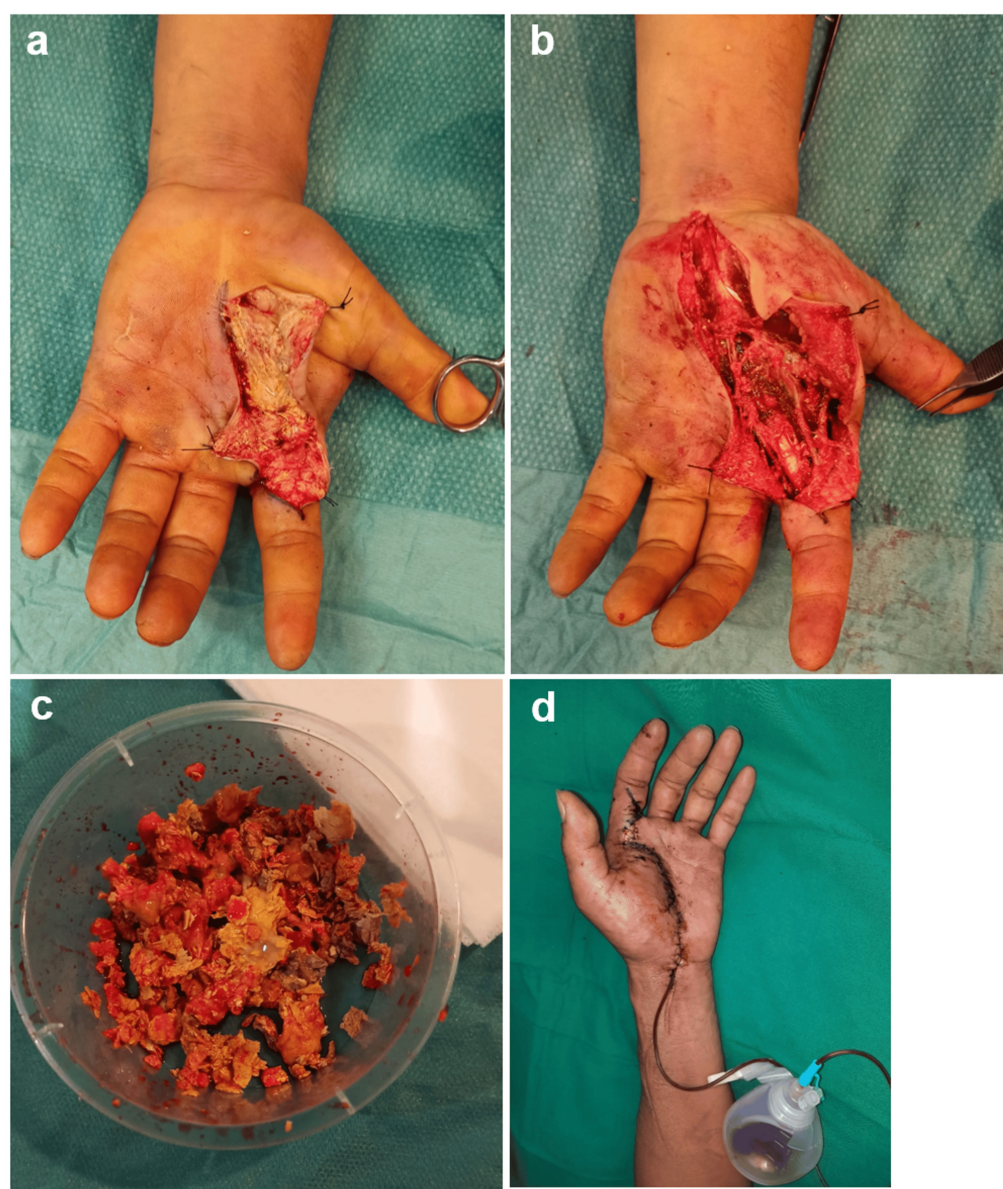
Case 3 (a) Intraoperative clinical aspect showing a significant amount of golden-yellow solid material along the thenar, mid-palmar, and dorsum of the hand at the levels of the second and third fingers, involving the flexor sheath and neurovascular bundles. (b) Intraoperative clinical aspect following product removal, neurolysis, and arteriolysis. (c) Multiple fragments of solidified material removed. (d) Immediate postoperative clinical aspect showing a suction drain in place.

Immediate surgical exploration revealed a significant amount of golden-yellow solid material along the thenar, mid-palmar, and dorsum of the hand at the level of the extensors of the second and third fingers and also involving the flexor sheath and neurovascular pedicles of the palm and second finger (Figure 4a). The product infiltrated and coated all discernible local anatomical structures. Multiple thrombosed dorsal veins were evident as well as signs of digital nerve injury in continuity. Access Bruner incisions allowed immediate decompressive fasciotomies in the thenar, mid-palmar, carpal tunnel, and dorsum of the hand compartments, followed by abundant irrigation and meticulous product removal from neurovascular bundles and tendons (Figure 4b, 4c). Microsurgical neurolysis and arteriolysis were performed, and the fingers exhibited adequate vascularization post-surgery. A suction drain was placed before closing the wound primarily (Figure 4d).

**Figure 5:**
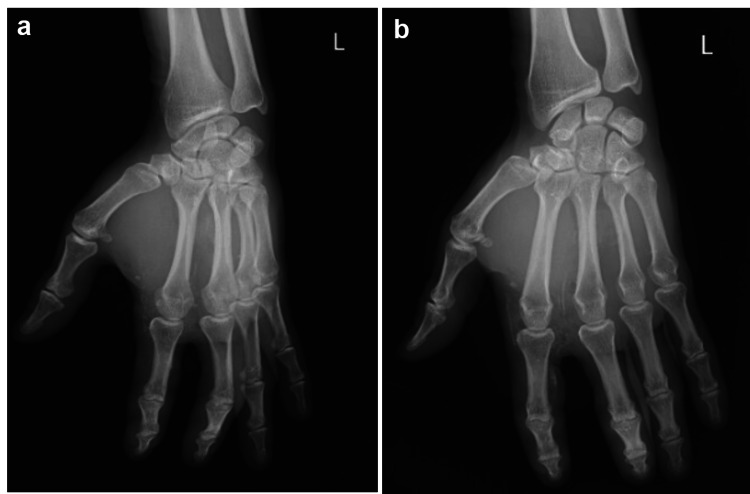
Case 3 (a, b) Postoperative X-ray of the left hand showing no residual material.

**Figure 6:**
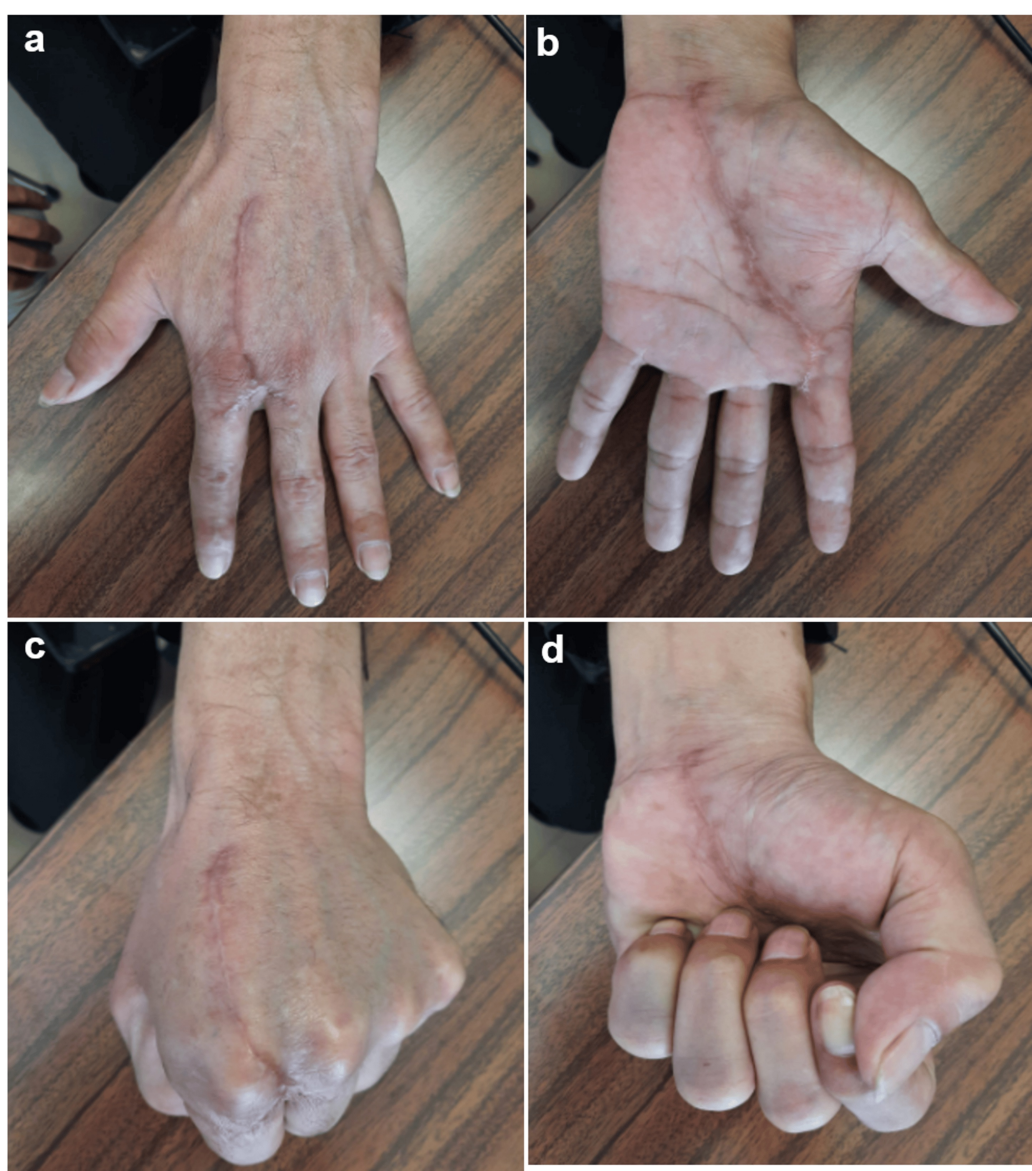
Case 3 (a, b, c, d) Clinical aspect six months postoperatively: complete flexion and extension of the fingers (plantar and dorsal views).

Postoperatively, the hand was placed in a palmar splint in the intrinsic plus position, and the patient received intravenous antibiotics for three days followed by oral antibiotics for eight days. The patient had a significant clinical improvement and was discharged on the third postoperative day for rehabilitation. Subsequent X-rays of the left hand showed no residual material (Figure 5a, 5b). At the six-month follow-up, the patient reported stiffness and hypoesthesia in the index finger, despite ongoing physical and occupational therapy. Full flexion and extension of the fingers were noted (Figure 6a, 6b, 6c, 6d).

## Discussion

High-pressure injected material spreads in an instant into the tendon sheath, along neurovascular bundles and fascial planes, following the path of least resistance [9]. Compression of the neurovascular bundles leads to vascular spasms, tissue ischemia, and thrombosis. If the tissue distension, caused by the injected fluid and subsequent swelling or edema, generates pressure exceeding hydrostatic pressure, it will restrict tissue perfusion, resulting in acute compartment syndrome and neurovascular compromise [3]. The hand palm has a larger expansion capacity than a finger; therefore, an injection with the same quantity of fluid at both sites results in the faster development of a compartment syndrome in the finger [3,10]. The injected material can extend to the forearm and even reach the mediastinum in extreme cases [11]. Injections into the thumb and small finger are more likely to extend into the forearm due to their connection with the radial and ulnar bursae, whereas injections into the index, long, and ring fingers tend to remain within the limited space of the tendon sheath. If the tendon sheath is penetrated over the thinner, membranous cruciate pulleys, the injected material may distend and destroy the tendon sheath [6].

In addition to the direct mechanical injury (high kinetic energy in a closed and poorly distensible anatomical compartment), some products are extremely cytotoxic and can cause chemical injury, tissue necrosis, and intense inflammatory responses, with the risk of secondary infection [7,10,12]. The type of injected product and its cytotoxicity are the most important prognostic factors in this type of injury [5,13]. Paints, solvents, paint thinner, plastic, lubricants, fuels, grease, cement, and hydraulic fluids are some of the substances that can be injected [6,11]. The injection of water, air, or small quantities of veterinary vaccine usually induces minimal inflammatory response and generally has a good prognosis. It can be managed non-surgically with close observation if compartment syndrome is not present [1,7,14]. On the other hand, turpentine and other organic solvents frequently used in an industrial context as diluents, dry cleaning, and paint thinners to oil-based paints are highly cytotoxic since they dissolve fats and lead to tissue liquefaction, offering an overall poor outcome and a 40% risk of amputation [1-3,5,6,13,15]. Solvents have a low viscosity and a fast distribution along the tissues [16]. The amputation rate is significantly higher following injuries involving oil-based paints than following water-based paints [5,17]. Other products like lubricants, wax, grease, and graphite usually do not provoke a very intense inflammatory response but can lead to chronic granulomas [6,13,15]. Secondary infection is often polymicrobial and occurs when tissue necrosis is present. Prophylactic antibiotic therapy is recommended to reduce the risk of infection [1,5]. Infection is relatively uncommon, because the injected material is usually an organic chemical that does not support bacterial growth [10]. Nevertheless, when a patient presents to the emergency department several days after the initial injury, suspicion should be raised, for example, in cases of flexor tenosynovitis. If the temperature of the injected solvent is very high, then it accentuates the injury by burning the underlying soft tissues and skin [18]. Wong et al. have proposed a clinical classification system to guide treatment [19]. The severity of the injury is divided into mild, moderate, and severe and is graded according to the nature of the fluid injected, the neurovascular status of the limb at presentation, and the delay until operative intervention. Mild injuries are considered for observation and conservative treatment, whereas moderate and severe injuries should be treated with emergent decompression and wide debridement [8,19].

This type of injury requires urgent assessment and treatment. It is important to assess the mechanism of injury, identify the type of substance involved, and determine the pressure level of the injection system to establish the prognosis and determine the most appropriate treatment. Moreover, it is crucial to exclude the potential for general intoxication, particularly with substances such as white spirit or turpentine [3,10]. Acute compartment syndrome constitutes a surgical emergency, with diagnosis primarily relying on clinical assessment; therefore, serial evaluations within the first hours after the injury are crucial, because even if not initially evident, it can develop due to inflammation and secondary edema. Preoperative plain radiographs can be helpful in defining the extent of infiltration if the injected substance is radiopaque, but treatment should not be delayed [13]. Radiopaque materials are easily visible on radiographs, and subcutaneous air is visible with radiolucent materials [8]. Some authors consider the latency time between the accident and the establishment of an adequate treatment the most important prognostic factor [1,2]. Not only the latency time to adequate treatment but also the injected fluid's nature, the pressure, the volume, and the location of injection have an influence on the seriousness and extensiveness of damage and functional outcome of the patient [3].

The amputation rate for this type of injury can be as high as 30-40% if not treated appropriately [3]. Immediate surgical treatment with decompressive fasciotomies, extensive washing and removal of the product, and aggressive surgical debridement plus carpal tunnel release, along with prophylactic antibiotics, tetanus prophylaxis, limb elevation, and palmar splint in intrinsic plus position for five days, has resulted in favorable clinical outcomes [1-3,6,8,19]. Ice and digital blocks should be avoided, and steroids are controversial [1,3,6,8]. Exploration under the ring block of the finger, use of the Esmarch bandage, removing the material with a solvent, and non-surgical fluid drainage should also be avoided [3]. Repeat debridements are usually necessary within 24-72-hour increments until the removal of all devitalized tissue and injected material is achieved. 

A rapid diagnosis, appropriate surgical intervention, ideally within six hours of injury [8], and postoperative rehabilitation through intensive physical and occupational therapy are essential in these injuries to enhance both the vital and functional prognosis of the limb, preventing severe complications and permanent sequelae, such as motor and sensory deficits, hyperesthesia, chronic pain, cold intolerance, chronic granulomas, and Volkmann's contracture of the hand [6,7]. Fibrosis arises around the tissues and can result in a strong restriction of the hand function. In some cases, revision surgeries may be necessary. Reconstruction by means of skin grafts and local or free flaps may also be necessary [19].

Implementing preventive measures for individuals operating high-pressure guns is important. These include education on the safe use of equipment, conducting regular functional checks, ensuring the use of protective clothing, and emphasizing the severity of hand injuries resulting from high-pressure incidents [1-3,20].

## Conclusions

High-pressure injection injuries of the hand presenting with acute compartment syndrome can have catastrophic consequences if not diagnosed and treated promptly. The cytotoxicity of the injected product and the latency time to adequate treatment are the most critical prognostic factors in this type of injury and will determine the potential sequelae for the hand. The severity and real extent of damage in high-pressure injection injuries are often hidden behind a small punctiform wound at initial presentation and are generally underestimated.

These types of injuries require urgent assessment, appropriate referral to a hand trauma center, and immediate surgical treatment, including decompressive fasciotomies and aggressive surgical debridement. Prompt diagnosis, early surgical intervention, and postoperative intensive physiotherapy are essential for hand salvage and function restoration.
